# Growth Hormone Deficiency and Treatment in Childhood Cancer Survivors

**DOI:** 10.3389/fendo.2021.745932

**Published:** 2021-10-22

**Authors:** Netanya I. Pollock, Laurie E. Cohen

**Affiliations:** ^1^ Division of Endocrinology, Department of Pediatrics, Boston Children’s Hospital and Harvard Medical School, Boston, MA, United States; ^2^ Dana-Farber/Boston Children’s Cancer and Blood Disorders Center, Boston, MA, United States

**Keywords:** growth hormone deficiency, growth hormone treatment, brain tumors, tumor recurrence, secondary neoplasm, childhood cancer survivors (CCS)

## Abstract

Growth hormone (GH) deficiency is a common pituitary hormone deficiency in childhood cancer survivors (CCS). The identification, diagnosis, and treatment of those individuals at risk are important in order to minimize associated morbidities that can be ameliorated by treatment with recombinant human GH therapy. However, GH and insulin-like growth factor-I have been implicated in tumorigenesis, so there has been concern over the use of GH therapy in patients with a history of malignancy. Reassuringly, GH therapy has not been shown to increase risk of tumor recurrence. These patients have an increased risk for development of meningiomas, but this may be related to their history of cranial irradiation rather than to GH therapy. In this review, we detail the CCS who are at risk for GHD and the existing evidence on the safety profile of GH therapy in this patient population.

## Introduction

Growth hormone deficiency (GHD) is the earliest reported and most common pituitary hormone deficiency in central nervous system childhood cancer survivors (CCS), with an overall prevalence of 12.5% (1). This is a consequence of the location of the brain tumor itself, as well as treatment modality, including neurosurgery, cranial radiation, and chemotherapy agents. Radiation therapy is an independent risk factor for development of GHD, and the risk increases with higher radiation doses to the hypothalamic-pituitary (HP) axis. In addition to impaired linear growth, children with GHD have been found to have reduced cardiac muscle mass, impaired lipid profiles, and increased fat mass (2, 3). Childhood cancer survivors with GHD have similar symptoms and comorbidities to those in the non-cancer population. Patients who receive craniospinal radiation are at even greater risk for short stature than those receiving cranial radiation alone due to direct damage of the spinal radiation on bone matrix, with the greatest deficit occurring in those irradiated at younger age (4). While the overall height benefit of growth hormone (GH) treatment is dampened in those who receive spinal radiation (5), those who are treated with GH still have a significant gain in height compared to those who are not. Additionally, treatment in adult cancer survivors with GHD has been shown to significantly improve overall quality of life (3, 6).

The long-term effects of GH treatment have been extensively studied, particularly the potential association with the development of malignancy, given the mitogenic properties of GH and its downstream target insulin-like growth factor-I (IGF-I) (7–11). This is of particular concern in CCS, as they are at higher risk *a priori* of tumor recurrence and secondary tumors. Previously, treatment with pituitary-derived GH in non-CCS was thought to be associated with increased incidence of colorectal cancer, new diagnosis of leukemia, and increased mortality from cancer overall (12–14). Since the advent of recombinant GH therapy, however, this link is not as clear (7–10). On the other hand, a French study reported an increased risk of all-cause mortality among patients treated with GH for low risk diagnoses (idiopathic GHD, short stature in those born small for gestational age, and idiopathic short stature) (15). These results, however, were from a preliminary study from only one of eight participating countries in the Safety and Appropriateness of GH treatments in Europe (SAGhE) study, a large cohort study of patients in 8 European countries treated with recombinant human GH for any indication between 1984 and 2007-2009, and were refuted in a second report from Belgium, the Netherlands, and Sweden (16). Therefore, the safety profile of GH remains an ongoing area of investigation, especially in CCS.

The aim of this review is to describe the sub-population of CCS at greatest risk for development of GHD and to highlight the most recent literature on the safety profile of GH therapy with respect to tumor recurrence and secondary malignancies in childhood cancer survivors.

## Defining Growth Hormone Deficiency in Childhood Cancer Survivors

The controversy in defining GHD in CCS is two-fold. Firstly, GHD is hard to diagnose, similar to the noncancer population. Secondly, some pediatric patients who have received cranial irradiation are thought to have appropriate stimulated GH levels but inadequate spontaneous secretion, a concept called neurosecretory dysfunction. In these cases, the HP axis is unable to generate sufficient levels of GH required in children for growth in puberty (17–19). This dysfunction has not been substantiated in adults (20), but suggests that cases of GH deficiency or insufficiency in CCS may go undiagnosed. This area remains controversial due to the difficulties in defining growth hormone insufficiency, detailed below. Further, evaluation of spontaneous GH secretion is not recommended due to overlap between these GH secretory profiles in healthy individuals and those with GHD, inconsistent results, and cost and burden to the patient (2, 21).

Commonly, IGF-I levels are used in the initial diagnostic evaluation of non-CCS due to ease of measurement given the stable levels throughout the day (versus the pulsatile pattern of GH). IGF-I levels may be reduced in malnutrition states or chronic diseases, and so IGFBP-3 may also be utilized (2). However, patients with normal stature and those with short stature without GHD can also exhibit IGF-I and IGFBP-3 levels below zero SD (22). The specificity of IGF-I and IGFBP-3 levels in GHD diagnosis is about 69% and 79%, respectively in non-CCS (23), with greater sensitivity and specificity at values less than -2 SDS (24).

Some studies have reported that IGF-I levels should not be used in CCS due to poor sensitivity of IGF-I levels in patients who have received cranial irradiation (24, 25), while others have found that IGF-I can still be used as a proxy for GH function (26). Cattoni et al. supported the use of IGF-I as a screening test for GHD, as they found a statistically significant correlation between IGF-I levels and GH peak in patients who have received cranial irradiation. Even though the overall sensitivity of IGF-I was low, there was diagnostic value when the IGF-I was found to be less than -2 SDS (27). While Weinzeimer et al. also demonstrated IGF-I as not being a sensitive screening tool in children with brain tumors, the majority of their patients diagnosed with GHD had an IGF-I level below 0 SDS (28) suggesting that an IGF-I level above 0 SDS makes GHD unlikely. IGBFBP-3 is an even less reliable indicator of GHD in the childhood cancer population, where levels can be normal or above – 2 SD in up to 50% of patients. Further adding to the diagnostic difficulty is the increase in IGF-I and IGFBP-3 levels during puberty, where some have normalization of levels despite abnormal GH secretion (28). Therefore, IGF-I and IGFBP-3 may not be the most reliable indicators of GHD in the CCS population, particularly after initiation of puberty.

The recommendations for diagnosis in CCS, therefore, are the same as in the non-CCS population, which include GH provocative testing with two agents, and against relying solely on IGF-I levels (2, 21). Previously, the gold standard for diagnosis of GHD was the insulin tolerance test (ITT), however, this may be dangerous in the pediatric population due to the severe resultant hypoglycemia (22). The threshold values for peak GH after provocative testing have varied between institution and studies, with some reporting partial GHD if peak is between 7-10 ng/mL and severe deficiency if stimulated peak is < 7 ng/mL (18), while others have used a cut off of GH sufficiency if levels are > 15 mU/L (5.775 ng/mL) (29) making comparison between studies difficult.

## Growth hormone and IGF-I signaling and links to cancer

Much of what we know about the side effects of GH and its role in cancer biology is through the study of patients with acromegaly with supraphysiologic and prolonged exposure to GH and IGF-I. Mortality rates are 2 to 2.5 times higher in patients with acromegaly, and with normalization of GH and IGF-1 levels, the mortality risk is similar to the general population (30, 31). Though controversial, patients with acromegaly were previously believed to have increased risk of cancer, particularly colorectal and thyroid cancer, as well as increased mortality from cancer overall (30, 32). A recent meta-analysis by Bolfi et al., however, demonstrated that upon control of disease (defined by normalization of IGF-1 level with varying cut-offs based on the study), the cause of death becomes similar to the general population, This analysis showed that the increased cancer incidence was for cancers not typically related to acromegaly, but instead those associated with environmental and genetic factors, as well as aging (31).

GH promotes cell proliferation, epithelial-to-mesenchymal transition, angiogenesis, and inhibition of apoptosis *via* activation of the janus kinase (JAK)/signal transducer and activator of transcription (STAT) and mitogen activated protein (MAP) kinase pathways (11, 33). The pro-tumorigenic effects of GH and IGF-I are counterbalanced by the IGF-II receptor and IGFBP-3, which have been shown to inhibit mitogenesis and stimulate apoptosis (33). *In vivo*, rats injected with pituitary-derived GH have increased number of neoplasms compared to controls, whereas knockout of the GH receptor in mammary cancer mouse models results in slower tumor growth (34, 35). Furthermore, high concentrations of IGF-I have been found in patients with prostate, colorectal, and breast cancers, suggesting that IGF-I level is correlated with risk of these cancers. The association of IGFBP-3 has not been as clear, but some have found levels to inversely correlate with risk (11, 33). It has thus been postulated that GH may promote an environment that is favorable for tumorigenesis (36), although may not be causative.

## Patients at Greatest Risk for Developing GH Deficiency

The GH axis is thought to be the most radiosensitive of the HP axes, which is why isolated GHD may occur at lower doses of radiation (17). **Tables 1**, **2** describe risk of GH abnormality based on radiation dose and malignancy type. As such, GHD is the most common pituitary dysfunction in CCS receiving radiotherapy. The deficit is thought primarily due to hypothalamic damage rather than pituitary, as patients with radiation induced GHD still exhibit appropriate stimulated response to GHRH analogs (37, 38). Risk increases with greater total dose, decreased number of fractions delivered to the hypothalamus and pituitary, and time since radiation. A 17.3% cumulative incidence of GHD after 15 years was noted in in the Childhood Cancer Survivor Study (CCSS) (39), and GHD was present in 46.5% of adult CCS observed for a mean of 27.3 years in the St. Jude Lifetime Cohort Study (40). GHD has also been described in patients receiving targeted immunotherapy with imatinib and ipilimumab (41).

**Table 1:** Radiation therapy and growth hormone deficiency in primary central nervous system tumors.

CNS Primary Malignancy	Radiation Dose	GH Abnormality	Studies
Non-pituitary brain tumors	30-50 Gy	Dependent on radiation schedule, age, length of follow-up and diagnostic thresholds; incidence may be lower if proton RT used	(42)
	> 37.5 Gy	87% at 2.5 years with GHD	
	<37.5 Gy	33% at 2.5 years with GHD	
Pituitary tumors or suprasellar region	24-56 Gy	Commonly GHD on presentation due to tumor location	
-Pituitary adenoma	35-45 Gy	Universal GHD within 5 years	(43)
-Suprasellar glioma/optic chiasmatic-hypothalamic glioma	45-55 Gy	Almost all within 2-3 years	(44, 45)
-Craniopharyngioma	54 Gy	In almost all (92%) following treatment (surgery +/- post- operative radiation)	(46)
-Germ cell tumor	24-36 Gy	Limited evidence on documented GH levels.	(47)
		Growth retardation on presentation with no new cases after RT	

CNS, central nervous system; Gy, gray; GH, growth hormone; GHD, growth hormone deficiency; RT, radiation treatment.

**Table 2:** Cranial radiation therapy and growth hormone deficiency in non-central nervous system tumors.

Non-CNS Primary Malignancy	Radiation Dose	GH Abnormality	Studies
Conditioning for hematopoietic stem cell transplant (leukemia, neuroblastoma)	7- 8 Gy single dose TBI	No GHD	(48, 49)
	10-12 Gy TBI	Isolated GHD in some	(43, 48–50)
CNS prophylaxis or CNS disease in acute lymphoblastic leukemia	18-24 Gy cranial radiation;	Pubertal GH insufficiency (reduced spontaneous GH secretion);	(51–54)
	12 Gy cranial radiation (for infants)	Compensated GHD (reduced stimulated but normal spontaneous GH levels);	
		Isolated GHD in < 30%	
Nasopharyngeal carcinoma and tumors of skull base	45-66 Gy cranial radiation	GHD in almost all adult patients (96.8%) in 5 years	(55, 56)
Retinoblastoma	Estimated 13-65 Gy to HP axis	30% with GHD;	(57)
		50% GHD with 20-30 Gy to HP axis	

Gy, gray; GH, growth hormone; TBI, total body irradiation; GHD, growth hormone deficiency; HP, hypothalamic-pituitary.

Isolated GHD can result following cranial irradiation with 18 or 24 gray (Gy) in pediatric patients with acute lymphoblastic leukemia (ALL) (18); patients treated with 24 Gy have significantly greater loss in height SDS than patients treated with 18 Gy (51). Some pediatric patients who undergo BMT for hematologic malignancy after conditioning with total body irradiation of 12 – 14.4 Gy have also been reported to develop GHD (50, 58).

Patients with primary CNS tumors receive higher doses to the CNS than patients with hematologic malignancies and so have greater risk of GHD, as well as additional anterior pituitary hormone deficiencies. Amongst pediatric patients who received an estimated 27 to 47.5 Gy to the HP axis for either CNS prophylaxis or a primary brain tumor outside of the HP region, 55% had GHD within 1 year (29). The time to onset was significantly shorter in those who received ≥ 30 Gy, and at > 5 years after radiation therapy, 100% of patients who received > 35 Gy had developed GHD (29).

Additionally, in a study of patients that included those with pituitary tumors or tumors in the region of the pituitary fossa, all patients (n=23) who received 35-45 Gy had developed GHD after 5 years of treatment (43). In a model by Merchant et al, it was suggested that GHD in pediatric patients with primary CNS tumors can occur as early as 12 months after initiation of cranial radiation therapy > 60 Gy, at 26 months with treatment doses of 25-30 Gy, and at 60 months with treatment doses of 15-20 Gy. Furthermore, in order for patients to have a <50% chance of GHD at 5 years, defined as peak GH below 7 ng/mL, the mean dose to the hypothalamus should not exceed 16.1 Gy over the course of 6 – 6.5 weeks (59).

With variation in the severity of GHD, the GH dose may also differ. Goal treatment IGF-I levels remain unclear (e.g., whether concern if above 0 SDS), but the consensus opinions for those undergoing GH treatment remain the same between the cancer population and non-cancer population and includes maintaining serum IGF-I levels within the normal range for age and pubertal status (2).

## Safety Profile of GH Replacement in Childhood Cancer Survivors

Childhood cancer survivors are at great risk of severe and life-threatening conditions, and by age 50 years, 22.5% of survivors have been shown to have two or more serious conditions (60–62). In an analysis of 14,358 patients in the CCSS, Armstrong et al. found that 1,382 patients (9.6%) developed one secondary neoplasm (SN), and 385 patients developed two SN (63). Risk factors included female sex, young age, radiation exposure, family history of cancer, and primary diagnosis of sarcoma or Hodgkin lymphoma (63).

To date, multiple large observational studies have sought to determine the risk of tumor recurrence and SN in patients treated with GH. These studies are limited by the different underlying conditions leading to GHD, difference in treatment exposures, small sample size, length of follow-up, and retrospective design. Additionally, there is lack of standardization of laboratory assays and diagnostic thresholds for GHD. Published studies do not routinely describe the severity of the GHD in the patient population or the doses of GH used. These challenges make it difficult to definitely state that there is no risk of tumor recurrence or SN, but the existing data do suggest that GH replacement in deficient individuals is likely safe. **Tables 3**, **4** describe these studies, where **Table 3** also includes patients treated with GH beyond the CCS cohort. Unfortunately the existing studies exclude those with cancer predisposition syndromes (e.g. Fanconi anemia, Bloom syndrome, Li-Fraumeni syndrome, Lynch syndrome) and so a consensus about GH treatment in this population does not exist. This population is of particular interest as they include disorders of DNA damage/repair, and as growth factors, GH and IGF-1 may reduce time for DNA repair by promoting progression of the cell cycle (76).

**Table 3:** Post-marketing or large observational cohort studies.

Firth author, year (ref)	Study Cohort	Total Cohort size, n	Number on GH, n	Mean age at GH therapy, y	Mean duration of GH therapy, y	Mean duration of follow-up, y	Person-years of follow-up, n	Primary endpoint	Comparison group	Conclusion
Savendahl, 2021 (64)	NordiNet international Outcome Study and ANSWER Program	37,702	37.702	9.7	3.5	Not mentioned	130,476	Long-term safety	Within NordiNet and ANSWER cohort	Incidence of adverse effects correlated with increased mortality risk category
						Up to 25 years				
Savendahl, 2020 (65)	SAGhE cohort (comprehensive)	24,232	24.232	10.5	5.0	16.5	400,229	Long-term overall and cause specific mortality	Within SAGhe cohort	Mortality not associated with mean daily or cumulative GH dose. Increased mortality risk in higher mortality risk diagnoses
Swerdlow, 2018 (66)	SAGhE cohort (Belgium, Netherlands, Sweden, UK)	10,403	10,403	Not mentioned	Not mentioned	14.9	154,795	Incidence of meningioma in GH treated patients	Within SAGhe cohort	Increased risk of meningioma following GH treatment. Greater risk in patients who received cranial radiation
Child, 2018 (67)	GeNeSIS	22,845	22,311	9.5	4.9	4.2	104,000	Standardized mortality radio and standardized incidence ratio for mortality, diabetes and primary cancer	Within the GeNeSIS cohort	Overall risk of death not elevated. Most common SN was meningioma; all patients with secondary meningioma received radiation.
Swerdlow, 2017 (8)	SAGhE cohort (comprehensive)	23,984	23.984	Not mentioned	Not mentioned	16.5 for mortality	396,344 for mortality	Cancer incidence and cancer mortality	Within the SAGhE cohort	Cancer risk unrelated to duration or cumulative GH dose
						14.8 for cancer incidence	154,371 for cancer incidence			
Carel, 2012 (15)	SAGhE cohort (France)	6,928	6,928	15.1 (at end of treatment)	3.9	17.3	116,403	All-cause and cause-specific mortality	Within the SAGhE cohort	Mortality rates increased, particularly in those with higher GH doses.
Savendahl, 2012 (16)	SAGhE cohort (Belgium, Netherlands, Sweden)	5,299	5,299	Not mentioned	Not mentioned	Not mentioned	46,556	Vital status, cause of death	Within the SAGhE cohort	Majority of deaths were due to accident or suicide.
Bell, 2010 (7)	NCGS	54,996	54,996	10.3 (age of enrollment)	3.6	Not mentioned	195,419 of treatment	Safety data and adverse events	Within the NCGS cohort	Overall safe profile. Craniopharyngioma was largest group of tumor recurrence.

ref, reference; GH, growth hormone; y, year; ANSWER, American Norditropin^®^ Studies: Web Enabled Research; SAGhe, Safety and Appropriateness of Growth hormone treatments in Europe; GeNeSIS, Genetics and NeuroEndocrinology of Short Stature International Study; NCGS, National Cooperative Growth Study.

**Table 4:** Childhood cancer studies.

First author, year (ref)	Study Cohort	Total Cohort size, n	Median age, y		Median duration follow up, y		Number on GH, n	Median age at start of GH, y	Median duration of GH therapy, y	Comparison group	Primary endpoint	Conclusion
Journy, 2021 (68)	FCCSS	7,670	6 (at primary cancer diagnosis)		Not mentioned		47	Not mentioned	Not mentioned	FCSS patients matched by sex, year of cancer diagnosis, and follow-up time	Clinical and therapeutic risk factors associated with CNS SN	GH therapy not associated with CNS SN. Secondary meningioma associated with radiation.
			29 (at subsequent cancer diagnosis)									
Thomas-Teinturier, 2020 (69)	Euro2K cohort	2852	GH treated	Non-GH treated	GH treated	Non-GH treated	196	10	4	Matched by radiation dose, gender, age at first diagnosis cancer, year of first diagnosis, and follow-up duration	Impact of GH treatment on SN	GH therapy does not increase risk of SN.
			4	4	26	27		
Woodmansee, 2013 (70)	GeNeSIS	421	5.4		2.1		394	Not mentioned	2.9	Within GeNeSIS, non-GH treated survivors	Incidence of SN in GH-treated childhood cancer survivors	Increased risk of SN in GH-treated childhood cancer survivors. Most common SN was meningioma.
	HypoCCS	280	8.4		2.9		244	Not mentioned	6.8	Within HypoCCS, non-GH treated survivors
Patterson, 2014 (71)	CCSS	12,098	Not mentioned		Not mentioned		338	Not mentioned	Not mentioned	Within CCSS	Incidence of CNS SN in GH-treated cancer survivors	No increase in risk of CNS-SN with GH treatment.
Mackenzie, 2011 (72)	Individual study	224	GH treated	Controls	14.5		224	Not mentioned	8.0	From radiotherapy database, matched by total radiation dose, age at diagnosis, duration follow-up, radiation target for primary tumor	Incidence of recurrence and SN after CNS irradiation	No increased risk for tumor recurrence or SN in GH-treated CNS survivors.
			33	29				
Sklar, 2002 (73)	CCSS	13,539	GH treated	Non-GH treated	6.2		361	10	4.6	Within CCSS, non-GH treated survivors	Risk of recurrence and SN in GH treated survivors	GH does not increase risk of tumor recurrence or death.
			3.5	7.2				
Ergun-Longmire, 2006 (74)	CCSS	14,108	GH treated	Non-GH treated	8.8		361	11	4.6	Within CCSS, non-GH treated survivors	Risk of recurrence and SN in GH treated survivors	GH-treated survivors have higher risk of SN than untreated. Most common SN was meningioma.
			3.5	7.1
Darendeliler, 2006 (75)	KIGS (Pfizer International Growth Database)	1,038	7.0 – 12.1 (varied by tumor type)		5.8 (patients without recurrence)		1,038	8.0 – 12.6 (varied by tumor type)	2.3 – 2.8 (varied by tumor type)	Within KIGS database (all patients treated with GH)	Risk of tumor recurrence based on tumor type	Recurrence highest in those with craniopharyngioma. Dose of GH did not differ between patients with and without recurrence.

FCCSS, French Childhood Cancer Survivor Study; GeNeSIS, Genetics and Neuro Endocrinology of Short Stature International Study; HypoCCS, Hypopituitary Control and Complication Study; CCSS, Childhood Cancer Survivor Study; Kabi International Growth Study (Pfizer International Growth Database); GH, growth hormone; y, year; CNS, central nervous system; SN, secondary neoplasm.

Sävendahl et al. recently reported results from two large multicenter observational studies, NordiNet International Outcome Study and ANSWER Program, two international pharmacoepidemiological registry studies sponsored by Novo Nordisk (64). The study population included 37,702 patients, 1,149 patients with history of neoplasm prior to GH treatment. In the 10 years of follow-up, 56 patients reported 62 neoplasms. However, of these 62 neoplasms, 59.7% were considered unlikely to be related to GH treatment, 35.5% were thought to be possibly related to GH treatment and only 4.8% were probably related to GH treatment. The neoplasms documented included both benign and malignant, and the group unfortunately does not differentiate between recurrent tumors and SN. Other adverse events reported in all three groups were similar to prior reports (7, 10). Notably, the incidence of adverse events correlated with increasing risk category, emphasizing the need for close monitoring of those with high-risk illnesses including CCS (64).

### Growth Hormone Therapy and Tumor Recurrence

One of the earliest individual studies to look at risk of tumor recurrence was a report in 1985 of 34 children with brain tumors, 24 of whom received GH. Thirty-three percent of patients treated with GH had tumor recurrence compared to 30% who did not receive GH (77). Due to the small nature of this study (less than 200 patients), it is not included in **Tables 3** or **4**. The similar incidence of tumor recurrence between treated and untreated groups has been supported by a number of other individual studies (72, 78–81). Mackenzie et al. compared 224 patients treated with GH to controls and found no significant difference in incidence of recurrent tumors (5.5% versus 7.3% respectively) (72). Mackenzie’s study was strengthened by matching the comparison group for age, sex, radiation dose and fractionation. Rohrer et al. reported the risk of CNS tumor recurrence in 108 children treated for craniopharyngioma, ependymoma, and medulloblastoma (79). Approximately 30% of patients (13 of 44) treated with GH had a recurrence and 47% (28 of 59) patients not treated with GH had tumor recurrence, concluding that there was no association with GH treatment (79). Bell et al. reported findings from the National Cooperative Growth Study (NCGS), an open-label multicenter, post-marketing surveillance study of approximately 55,000 patients sponsored by Genentech. Of the 2,500 patients with non-pituitary CNS or extracranial malignancy prior to treatment, 99 patients had recurrent non-pituitary CNS tumors and 24 patients had recurrence of extra-cranial malignancy. The largest single group of intracranial tumor with recurrence was craniopharyngioma, with 8.7% of patients with craniopharyngioma recurrence (7). This study is limited by the inability to compare recurrence rates to those who were not treated with GH.

Sklar et al. were able to make comparisons in recurrence rates between GH-treated and untreated patients in the CCSS. They followed patients for a median time of 6.2 years and found 354 patients who were treated with GH (172 with primary brain tumors) amongst 13,539 total participants (73). For all patients with previous cancer diagnoses, the risk of disease recurrence was not significantly greater for those treated with GH compared to those who were not treated (RR 0.83). However, for patients with brain tumors, risk of disease recurrence was significantly reduced compared to survivors not treated with GH (RR 0.31), perhaps owing to patients with better prognosis selected for treatment with GH. Darendeliler et al. further details the recurrence rates by type of CNS tumor in the Kabi International Growth Study (KIGS) database, a Pfizer post-surveillance observational investigation of 1038 patients with CNS tumors who were treated with GH (75). Frequency of recurrence was 11.7% amongst patients with craniopharyngioma, 4.7% amongst patients with medulloblastoma, 8.8% in ependymoma group, 4.0% in germinoma, and 9.8% in glial tumors. Comparison in recurrence rates between patients who were not treated with GH was not made. There was no difference in dose of GH between patients with and without tumor recurrence (75).

### Growth Hormone Therapy and Secondary Neoplasms

In the aforementioned study by Sklar et al., there were 15 subsequent neoplasm (SN) that occurred after start of GH therapy, all of which were solid tumors, and 14 occurred at a site previously exposed to external radiation or after treatment with alkylating agents. They reported an increased risk of SN in GH-treated survivors, with a high number of SNs in patients with primary acute leukemia. However, when looking at malignant SN only (excluding meningiomas), the increased risk in GH-treated survivors diminished (73). This same cohort was followed for an additional 32 months with Ergun-Longmire et al. reporting 5 additional solid tumors for a total of 20 SN (74). There were no cases of subsequent leukemias. Among 13,747 patients not treated with GH, 555 SN were reported. The significant risk factors for development of SN were age at diagnosis, use of alkylating agent, radiation therapy, and GH therapy. However, when comparing patients with the same original cancer diagnosis, there was no statistically significant difference between those who had received GH and those who had not (74). In this follow-up report of GH-treated survivors, meningioma was the most common SN (9 of 20), and all patients who developed meningioma had received cranial irradiation. There was not a statistically significant increased risk of death for GH-treated patients compared to those who were not treated (RR 1.2) (74). A subsequent report of the CCSS cohort focused solely on secondary CNS neoplasms and found that GH treatment was not associated with increased overall risk (RR 1.0). Risk factors for development of secondary CNS neoplasms, including both meningioma and glioma, included increased time since cranial radiation (71).

This increased incidence of meningiomas in GH-treated patients has been replicated in several recent large studies, including the SAGhE cohort and Eli Lilly’s GeNeSIS study (66, 67). The final results of the SAGhE study, reported in 2020 by Savendahl et al., showed that higher mortality risk after GH treatment was related to pre-existing mortality risk based on underlying disease (65). Preliminary reports published from the individual countries have reported on specific adverse events, including risk of new neoplasms (8, 15, 16).

In 2018, Swerdlow et al. studied 10,403 GH-treated patients from 5 of the 8 study countries and found that the standardized incidence risk (SIR) for meningioma was 75.4 (95% CI, 54.9 – 103.6) when compared with year-specific rates from the general population matched for sex, age, and country. This effect was primarily due to the higher risk of meningioma amongst those who had a prior diagnosis of cancer (SIR 466.3; 95% CI, 33.7-643.5) (66). There were 38 total meningiomas; 37 occurred in patients who received cranial irradiation. There was no significant association between risk of SN and mean GH dose, duration of treatment, or cumulative dose of GH (66).

The GeNeSIS study was a prospective observational program of 22,845 patients in 30 countries treated with GH between 1999 and 2015 (67). Amongst this patient population, there were 622 GH-treated CCS. In a study of final safety outcomes in 2018, Child et al. reported that the crude incidence rate (95% CI) of SN amongst the GH-treated childhood cancer survivors was 10.69 (13.3-21.47)/1,000 people years (67). Thirty-one patients had a SN (5.0%), and 25 of these patients had received radiation therapy during treatment for their primary neoplasm. The most common SN was meningioma and all patients with subsequent meningioma had previously received cranial irradiation (67). These studies suggest an association of GH treatment and SN amongst CCS, however, this is confounded by the established increased risk of meningiomas in those with prior brain radiation treatment (82).

Journy et al. report the risk specifically of subsequent CNS tumors in the French Childhood Cancer Survivor Study (FCCSS) (68). The FCCSS includes 7,670 patients diagnosed between 1946-2000 with a solid tumor or lymphoma (68). The risk of subsequent meningioma was 16 times higher amongst patients with prior CNS tumor than other cancer diagnoses, and the risk was significantly associated with cumulative radiation dose. However, there was no association of GH therapy with development of subsequent meningioma (68). Thomas-Teinturier et al. made similar conclusions in a 26 year follow-up of 2,852 patients identified from a separate French cohort of survivors from childhood solid malignancies (69). One-hundred and ninety-six patients in this group received GH treatment. A total of 374 patients (13%) developed at least one SN, with 40 SN occurring following GH treatment. There were none identified with subsequent leukemia, a finding that had previously been reported (74). Of the 40 SN, there were 17 meningiomas and 9 glial tumors. There was a significantly higher crude incidence of SN in GH-treated patients than untreated patients, however when adjusting for radiation dose and alkylating therapy, this difference disappeared. In multivariate analysis, GH treatment was not associated with increase in SN risk (RR 1.2, 95% CI 0/9-2) or meningioma occurrence (RR 1.9. 95% CI 0.9-4) (69).

## Summary/Discussion

Growth hormone deficiency may develop after radiation therapy that includes the HP region, with higher doses of radiation associated with increased likelihood of GHD and earlier onset. There are theoretical concerns about malignancy development if GH therapy is initiated if GHD is diagnosed in the CCS. However, the association between GH therapy and cancer has been extensively investigated, and while many existing studies are limited by lack of a comparison group, the current data suggest that GH therapy does not increase tumor recurrence in CCS. Some studies have suggested an increased risk of SN after GH therapy, the most frequent being meningiomas, but meningiomas are highly associated with radiation exposure irrespective of GH therapy. Overall, the safety data from studies are reassuring, but there are limitations. As long-term data are unknown, the risk vs. benefit ratio should be determined for every individual patient. Additionally, as our knowledge of the role of genetics in cancer development increases, an unanswered question is whether there is increased risk of SN during or after GH therapy in individuals with cancer predisposition syndromes.

## Author Contributions

NP and LC drafted the initial manuscript and revised for accuracy. All authors approved final manuscript as submitted.

## Conflict of Interest

LC has received honoraria for lectures for symposia that received educational grants from Novo Nordisk.

The remaining author declares that the research was conducted in the absence of any commercial or financial relationships that could be construed as a potential conflict of interest.

## Publisher’s Note

All claims expressed in this article are solely those of the authors and do not necessarily represent those of their affiliated organizations, or those of the publisher, the editors and the reviewers. Any product that may be evaluated in this article, or claim that may be made by its manufacturer, is not guaranteed or endorsed by the publisher.
